# Relationships of Community and Individual Level Social Capital with Activities of Daily Living and Death by Gender

**DOI:** 10.3390/ijerph13090860

**Published:** 2016-08-29

**Authors:** Haruhiko Imamura, Tsuyoshi Hamano, Takehiro Michikawa, Fujimi Takeda-Imai, Takahiro Nakamura, Toru Takebayashi, Yuji Nishiwaki

**Affiliations:** 1 Department of Environmental and Occupational Health, School of Medicine, Toho University, Tokyo 143-8540, Japan; f@e-mai.jp (F.T.-I.); md13032n@oc.toho-u.ac.jp (T.N.); yuuji.nishiwaki@med.toho-u.ac.jp (Y.N.); 2 Institute of General Education, Kyoto Sangyo University, Kyoto 603-8555, Japan; thamano@cc.kyoto-su.ac.jp; 3 Environmental Epidemiology Section, Centre for Health and Environmental Risk Research, National Institute for Environmental Studies, Ibaraki 305-8506, Japan; tmichikawa@nies.go.jp; 4 Department of Preventive Medicine and Public Health, School of Medicine, Keio University, Tokyo 160-8582, Japan; ttakebayashi@a3.keio.jp

**Keywords:** Activities of Daily Living, death, social capital, participation, trust

## Abstract

This study determined whether there is an association between social capital and a composite outcome of decline in Activities of Daily Living (ADL) and death by gender. A prospective 3.5 year cohort study was conducted in a rural town in Japan. The study participants were 984 individuals aged 65 years and older with not impaired on ADL at 2010 baseline survey. Social participation and generalized trust were measured as social capital. The individual level responses were dichotomized and aggregated into the community level (eight areas). Multilevel logistic regression adjusting for covariates revealed that social participation at the individual level was significantly associated with higher odds of composite outcome (OR of “not participate” = 1.97, 95% CI = 1.38–2.81). Regarding generalized trust, only in men, there was an inverse association at the community level (OR of “low” = 0.55, 95% CI = 0.32–0.96), and a positive association at the individual level (OR of “tend to be careful” = 2.22, 95% CI = 1.27–3.90). These results suggest that social capital were associated with a decline in ADL and death and that the association may differ by gender.

## 1. Introduction

There have been great efforts to identify the effect of social capital on health. Putnam explains social capital as “features of social organization, such as trust, norms, and networks that can improve the efficiency of society by facilitating coordinated actions” [1]. Although there were conflicting definitions of social capital, Putnam’s framework provided a new empirical base for social capital theory and was introduced to the public health field [2]. In addition, some forms and dimensions of social capital were proposed recently. Among them, social capital can be broken down into two aspects: structural and cognitive [3]. The structural aspect indicates community or organization based formal/informal composition, practices, and fields of activity (including “networks”). The cognitive aspect indicates the relevant individual’s values, attitudes, codes, and beliefs (including “trust” and “norms”) [3,4,5]. Because many previous studies used both aspects [4], using this framework could provide comparable results.

Furthermore, social capital can be summarized into two approaches: considering it as resources embedded in the social network focusing on personal characteristics [6] and considering it as “social cohesiveness” [7]. The latter is especially characterized by its focus on contextual effect [8], implying that collective characteristics, such as community and social organization level practices and rules, influence the individual. In recent studies, the influence of both personal characteristics and contextual effects has been observed with a multilevel analysis [9]. Although the majority of the outcomes in previous studies were self-rated health [10,11,12,13,14,15,16,17], mental health [12,18,19,20], and death [21,22,23,24], few studies focused on a decline in Activities of Daily Living (ADL) as the outcome [25,26]. The maintenance of ADL is related to Quality of Life in older persons. 

Theoretically, social capital affect health outcomes through several plausible pathways: (1) influencing health-related behaviors (promoting more rapid diffusion of health information or increasing the likelihood that healthy norms of behaviours such as physical activities are adopted, and exerting social control over deviant health-related behaviors); (2) influencing local access to local services and amenities such as recreational facilities; (3) affecting psychosocial process [8]. Particularly, with regard to ADL, the former two may be relevant. In addition, previous studies showed the relationships between social capital and physical activities [27,28]. Because physical activities are associated with ADL [29], social capital may affect ADL through physical activities.

In addition, gender differences have not been fully argued in the debate on social capital and health research. A recent study reported that a gender difference exists in the social networks, especially in terms of composition, quantity, and type [20]. Several studies suggested that gender difference is also present in the influence of social capital on health [13,24,25,30,31,32,33]. For example, there were the positive association between individual level participation and self-rated health [13] or death [24] in men, community level trust and self-rated health [33] or ADL [25] in women, community level trust and self-rated health [32] in men. Consistent results however have not yet been obtained. The aim of this cohort study focused on an older persons living in a community was to determine whether there is an association between social capital at the community and individual level and a composite outcome of decline in ADL and death by gender.

## 2. Methods

### 2.1. Study Population

**Figure 1:**
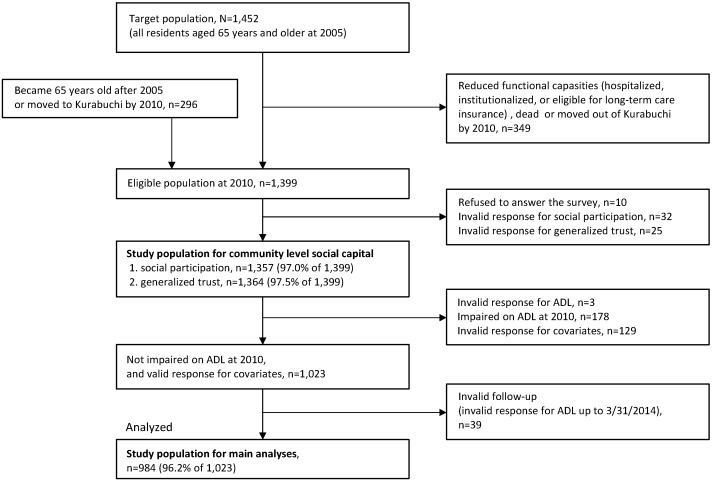
Study population.

This study was part of the Kurabuchi study [34,35,36] that has targeted all 1452 residents aged 65 years and older in Kurabuchi town (Takasaki City, Gunma Prefecture, Japan) since 2005. After the baseline health survey in 2005 was carried out, follow-up surveys were continued annually. In the follow-up 2010 survey, the item of social capital was included into the questionnaire. It is for this reason that we used the 2010 survey as a baseline data in the present study. In the 2010 survey, we excluded residents who had already experienced a decline in function (e.g., hospitalized, institutionalized, or eligible for long-term care insurance), and identified 1399 residents aged 65 years and older as eligible population; 1103 residents who were the participants of the 2005 survey, and 296 residents who became 65 years old after 2005 or had moved to Kurabuchi town after 2005. The postal questionnaire was distributed to each home of the eligible population and collected by trained nurses and local welfare workers with the cooperation of the town. Ten participants refused to respond, and valid response to a questionnaire on social capital were collected (1364 valid responses for social participation and 1357 valid responses for generalized trust, respectively). Of those, 1023 were not impaired on ADL as assessed by the Katz Index of ADL [37] and had valid responses for all covariate items. Follow-up ADL data were obtained from the annual survey until 2013. After excluding 39 individuals with invalid responses for follow-up ADL, 984 (96.2% of 1023) were analyzed (Figure 1).

This study was approved by the Ethics Committees of Keio University School of Medicine, Tokyo, Japan (No. 16-20) and the School of Medicine at Toho University, Tokyo, Japan (No. 23046, 2700623046).

### 2.2. Outcome Measurements

We defined composite outcome including decline in ADL and death. Decline in ADL was defined as either admission to a nursing home or need of assistance at home during the follow-up period. The latter was defined as long-term care (LTC) eligibility or a need for help in any of the six basic ADL items (bathing, dressing, toileting, transferring, continence, and feeding) in the Katz Index. LTC eligibility is a requirement for receiving LTC insurance services (such as home help, day service, institutional service and so on) in Japan, which began in 2000. Every Japanese person aged 65 and older is eligible for LTC insurance benefits and the service eligibility is determined based on nationally standardized needs certification system using the computer-aided assessment [38]. In this study, any of the seven levels of LTC insurance service was considered LTC eligible. Information on death, nursing home admission, and LTC eligibility was obtained from the Kurabuchi Branch Office of Takasaki City Hall until 31 March 2014.

### 2.3. Social Capital

In this study, social participation and generalized trust were measured to capture structural and cognitive social capital. These were typical indicators used in previous studies [4,18,25]. Each item was assessed at individual level and community level. Individual level social capital was assessed for the study population for main analysis (*n* = 984). We aggregated individual responses of social capital (*n* = 1357 for social participation and 1364 for generalized trust) into community level social capital using the baseline data. In Kurabuchi town, there are eight areas, which have the same range as a neighborhood association or senior citizens club, and community level social capital was evaluated in each area level.

Social participation was measured by the active members of a group/organization in the following four categories: (1) local community groups; (2) sports, hobby, or leisure group; (3) voluntary organization or non-profit organization; or (4) other organization. These categories were used in the previous study [13,26,39]. Response alternatives were “do not participate” and “participate”, the latter composed of six-stage frequency from “a few days a year” to “four or more days a week” for each group/organization. Responses were dichotomized into “participate; participate in one or more group/organizations regardless of the frequency” versus “not participate; do not participate in all group/organizations” and used as the individual level social participation. The percentage of “participate” was calculated for eight areas in the town and dichotomized as “high (top four areas)” or “low (lower four areas)” as the community level social participation.

Generalized trust was assessed by the following question: “Generally speaking, would you say that most people can be trusted, or that you cannot be too careful?”. This question was used in the previous studies [18,39]. Response alternatives were on a nine-point scale from “1; people can be trusted” to “9; you can’t be too careful” via “5; Middle” and “Unknown”. Responses were dichotomized into “tend to trust; 1–4” versus “tend to be careful; all other responses” and used as the individual level generalized trust. In order to measure in a more positive meaning, “middle” was not included in “tend to trust”. Then, the percentage of “tend to trust” was calculated for each area and dichotomized as “high (top four areas)” or “low (lower four areas)” for community level generalized trust.

### 2.4. Covariates

Age, sex, marital status, educational attainment, number of people living together, and self-rated health were obtained from the questionnaire as covariates. Sex, age, number of people living together, and self-rated health was obtained from the 2010 survey, and marital status and educational attainment from the 2011 survey. All variables except for age were dichotomized: sex (men vs. women); marital status (married vs. widowed, divorced, or single); educational attainment (≥10 years vs. <10 years); number of people living together (≥1 person vs. none); and self-rated health (very good, good, or fair vs. bad or very bad).

### 2.5. Statistical Analysis

Multilevel logistic regression models were created to analyze whether community and individual level social capital (social participation and generalized trust) at baseline were associated with the composite outcome of decline in ADL and death over the 3.5 year follow-up period. We created the following three multilevel models: First, community level social participation and trust were included in the model adjusting for age and sex (model 1). Then, individual-level social participation and trust were added to the model (model 2). Finally, marital status, educational attainment, number of people living together, and self-rated health were added to the model (model 3). Gender-stratified analyses were also conducted on all models to investigate gender differences in the effects of social capital.

Eight areas might be too few to perform multilevel analysis, however, because this study used the dataset with a hierarchical structure, we performed multilevel analysis which provided a robust standard error and confidence interval [40].

The following three sensitivity analyses were conducted on all models: first, the analyses were repeated after excluding deaths (*n* = 32) in order to remove the effects of competing risks associated with death. Second, we similarly used a study population excluding those who had decline in ADL or were dead in the first year of the follow-up period (*n* = 82) to remove the effect of reverse causation. Third, the analyses considering a history of major diseases as covariates were conducted using as a study population those who took a medical checkup and answered questions about medical history in 2009 or 2010 (*n* = 439). History of major diseases was defined as having any one of the following diseases known to be causes of death or disability in older adults: stroke, myocardial infarction/angina, diabetes, Parkinson’s disease, or cancer. The statistical significance level was set at *p* < 0.05. All analyses were performed with STATA version 13 (STATA Corporation, College Station, TX, USA).

## 3. Results

The mean age of the study population (*n* = 984) was 75.6 years (SD ± 7.0), 46.6% were men, and 21.3% had composite outcome (18.1% had decline in ADL and 3.3% were dead) over the follow-up period. Table 1 stratifies the baseline characteristics according to the composite outcome. The participants who experienced the composite outcome were older, not married, less educated, and less likely to participate than those who did not. 

Table 2 shows the area characteristics: Areas B, C, D, and E were classified as “high” and areas A, F, G, and H as “low” for social participation. Areas C, D, F, and H and areas A, B, E, and G were classified as “high” and “low” for generalized trust, respectively.

Table 3 shows the association of community and individual level social capital with composite outcome. In total, individual level social participation was significantly associated after adjusting for all other covariates (OR of “not participate” = 1.97, 95% CI = 1.38–2.81 in model 3). With regard to trust, low community level generalized trust was inversely associated with the composite outcome (OR of “low” = 0.62, 95% CI = 0.43–0.87 in model 3), though individual level generalized trust was not significantly associated. However, there was a clear gender difference in the association between individual level generalized trust and the composite outcome (*p* for interaction = 0.02). Men who tended to be careful were more likely to experience the composite outcome (OR of “tend to be careful” = 2.22, 95% CI = 1.27–3.90 in model 3), while low community level generalized trust was inversely associated with the composite outcome (OR of “low” = 0.55, 95% CI = 0.32–0.96 in model 3). In contrast, such an association was not observed in women. There was no statistically significant interaction between community and individual level social capital (*p* for interaction = 0.34 for social participation, and 0.83 for generalized trust in model 2).

Sensitivity analyses also shows similar results; excluding the deaths, excluding those who had the decline in ADL or were dead in the first year of the follow-up period and considering a history of major diseases as covariates (Tables S1–S3). 

**Figure 2:**
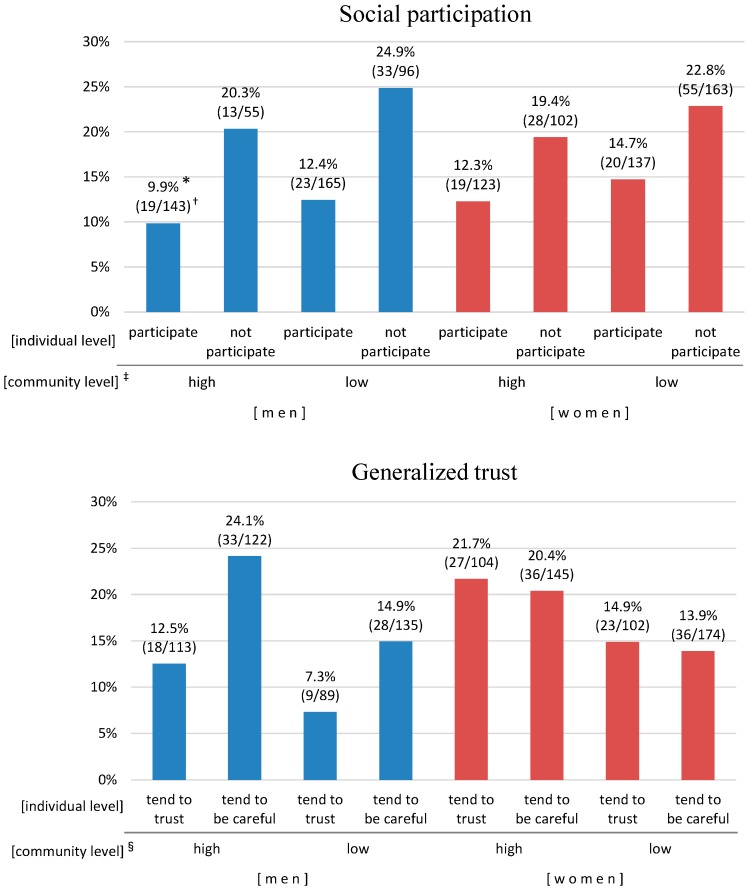
Relationship of community level and individual level social capital to composite outcome by gender. * Adjusted probability from the fixed effects only of composite outcome for each category by gender stratified multilevel logistic analyses after adjusting for all covariates; **^†^** Number of outcomes/analyzed population; **^‡^** High: area B, C, D, E. Low: area A, F, G, H; **^§^** High: area C, D, F, H. Low: area A, B, E, G.

Figure 2 shows the relationship of community and individual level social capital to composite outcome; adjusted probability from the fixed effects only of composite outcome for each category by gender-stratified multi-level logistic analyses in model 3. 

For social participation, in both men and women, those in the “not participate” group at the individual level had nearly two times more outcomes than those in the “participate” group regardless of the community level. For generalized trust, in each of the individual levels, those who were in the “high trust” area had many more outcomes than those who were in the “low trust” area. In addition, among men, those who in the “tend to be careful” group at the individual level had nearly two times more outcomes than those who in the “tend to trust” group.

## 4. Discussion

Our findings showed that non-participation at the individual level was associated with a decline in ADL and death in both men and women, while the social participation at the community level did not show a similar association. For generalized trust, there was a significant association with a decline in ADL and death, especially in men. Lower trust at the individual level and higher trust at the community level increased the odds of a decline in ADL and death.

Previous studies indicated that social participation at the individual level had a positive impact on self-rated health [13,17,31], mental health [18,30], and death [24], whereas the contextual effect of social capital was more weaker [12,17,18,23]. With regard to the impact on ADL, our results even after taking into consideration of both the levels simultaneously are consistent with previous studies [25,26], though previous studies considered only either community level [25] or individual level [26] social participation. It is hypothesized that participation to the community increases opportunities to build social networks which provide the access to local resources [8,27], and encourages physical activities [27] which associated with ADL [29]. Considering social participation had a positive impact in both men and women, it may be important to encourage the participation to the community regardless of gender.

As for generalized trust, results at the individual level in men were consistent with previous studies, which indicated the positive association with self-rated health [11,12,14,16,17] and mental health [12,18,20]. It is hypothesized that low trust toward others reduces opportunities to obtain information on health promotion through personal relationships, otherwise to receive administrative or social support, which may prevent the risk of a decline in ADL and death. With regard to trust at the community level, although our results did not support previous findings [12,15,18,25,32,33], a growing recognition of the “dark side” of social capital has stimulated in recent years [7,11,14,17]. For example, Campos-Matos et al. reported that people with lower levels of trust at the individual level showed lower self-rated health in communities with higher country-level trust [14]. We also tested interaction, however there was no statistically significant between community and individual level social capital. The reason why generalized trust at community level could not have positive impact on health is uncertain. It might be explained that those who live in a community with higher levels of trust, where mutually monitoring and interfering relationships are not routinely performed, have an indifference toward people with a health problem, including ADL decline or death. If this hypothesis is supported, it is necessary to conduct personal assessments to thoroughly provide public support, such as caring for people at high risk even if they are part of a community with higher social capital.

In our study, gender difference in the influence of generalized trust at the individual level on health was observed. The possible explanation for this result may be related to Japanese culture. This can be hypothesized that weaker relationship, not strong but moderate, is more suitable for men to promote their health in the community. This is because networks for men are mainly and strongly formed through their work [41], and retired men tend to decrease the frequency of social contacts [42]. Previous studies reported that men are likely to participate formal organizations, while women are likely to engage in community action [13,20,43] and form local social networks that connect families and communities [20,32,44]. Retired men who have low trust may feel burden or stress to build and manage their new relationship in the community, especially in a high trust community. Thus, to make weaker relationship with neighbours in the community could be important for men before their retirement. Further study is needed to examine this association according to the longer period follow-up data.

This study has two major strengths. First, we achieved a high response rate and higher quality of follow-up (only 39 participants were lost to follow-up). Second, social capital at the community level was developed based on the data from overall study population that included the participants with decreased ADL at baseline. In the previous studies, social capital at the community level was developed by the aggregated data from the participants in the analysis population. Therefore, our social capital at the community level captured more accurately than the previous studies.

This study also has certain limitations. First, it is necessary to carefully consider the possibility of reverse causation. Particularly, it is crucial in the association between social participation and ADL. It might be thought that older persons who showed signs of ADL decline at baseline might become less willing to participate in the community. We conducted the sensitivity analyses excluding those experienced the decline in ADL or death in the first year of the follow-up period and the results showed a similar tendency (Table S2). However, odds ratio of “not participate” was decreased (1.55 in Table S2 and 1.97 in Table 3). These results might suggest that the possibility of reverse causation could not be completely denied. Second, although our study was a prospective cohort study, the follow-up period was moderately short. Considering the first limitation of the reverse causation, analyses with a longer follow-up period are necessary in the future. Third, this study was conducted in a single rural town. In addition, there was a possibility of the selective attrition of respondents between the target population at 2005 and the eligible population at 2010. The excluded respondents by 2010 (*n* = 349, Figure 1) might have lower levels of social capital. Therefore, the results of this study might be obtained mainly from the persons who had higher levels of social capital. Further study is needed to examine the generalizability of these findings. Fourth, in this study, social capital at the community level were measured by aggregating individual responses. This measurement might not capture the dynamics embedded in the concept of social capital. Indeed, there are arguments about the measurement of social capital at community level. Though there is alternative idea of using objective indicators representing social capital, appropriate indicators has not been established at present [4]. In addition, Subramanian et al. reported that neighborhood difference in trust measured by aggregating individual responses remain after adjusting individual-level socioeconomic status [40,45]. Many previous studies generally used aggregate measurement [4,7]. Fifth, with regard to the results of trust at the community level, selection bias should be considered. The person who should have outcome has already had it by the baseline of the study in a community with lower trust. In contrast, a community with higher trust tends to include many residents at high risk of decline in ADL and those residents experience the outcome occurrence during the follow-up. In fact, the proportion of those who impaired on ADL at baseline for eligible population was lower in the area with high trust (12.5% in area with “high trust”, and 14.2% with “low trust”). However, there was no statistically significant difference (*p* = 0.38). Further studies are necessary in this regard.

## 5. Conclusions

Non-participation at the individual level was associated with a decline in ADL or death in both men and women, while social participation at the community level did not show a similar association. Lower trust at the individual level and higher values of trust at the community level were associated with increased risk of a decline in ADL or death, especially in men. These results suggest that social capital is associated with a decline in ADL and death and that the association may differ by gender.

## Figures and Tables

**Table 1:** Baseline characteristics of the study population.

Characteristics	Total, *n* = 984 *n* (%)	Composite Outcome *		
		No, *n* = 774 *n* (%)	Yes, *n* = 210 *n* (%)	*p*-Value ^†^
**Sex**				
women	525 (53.4)	403 (52.1)	122 (58.1)	0.12
men	459 (46.6)	371 (47.9)	88 (41.9)	
**Age (years)**				
65–69	232 (23.6)	217 (28.0)	15 (7.1)	<0.001
70–74	227 (23.1)	203 (26.2)	24 (11.4)	
75–79	243 (24.7)	200 (25.8)	43 (20.5)	
80–84	152 (15.4)	101 (13.0)	51 (24.3)	
≥85	130 (13.2)	53 (6.8)	77 (36.7)	
**Marital status**				
Married	669 (68.0)	554 (71.6)	115 (54.8)	<0.001
Widowed, divorced, single	315 (32.0)	220 (28.4)	95 (45.2)	
**Educational attainment (years)**				
≥10	316 (32.1)	275 (35.5)	41 (19.5)	<0.001
<10	668 (67.9)	499 (64.5)	169 (80.5)	
**Number of people living together**				
≥1 person	853 (86.7)	671 (86.7)	182 (86.7)	0.99
None	131 (13.3)	103 (13.3)	28 (13.3)	
**Self-rated health**				
Very good, good, normal	905 (92.0)	717 (92.6)	188 (89.5)	0.14
Bad, very bad	79 (8.0)	57 (7.4)	22 (10.5)	
**Social participation**				
Participate	568 (57.7)	487 (62.9)	81 (38.6)	<0.001
Not participate	416 (42.3)	287 (37.1)	129 (61.4)	
**Generalized trust**				
Tend to trust	408 (41.5)	331 (42.8)	77 (36.7)	0.11
Tend to be careful	576 (58.5)	443 (57.2)	133 (63.3)	

* Decline in ADL or death; **^†^** Chi-square test.

**Table 2:** Area characteristics.

Area	Mean Age of Eligible Population (Years)	Community Level Social Participation (%) *	Community Level Generalized Trust (%) ^†^	Composite Outcome/Study Population for Main Analyses (%)		
				Total	Men	Women
A	76.4	76/170 (44.7) L	54/179 (30.2) L	20/123 (16.3)	6/54 (11.1)	14/69 (20.3)
B	75.6	82/143 (57.3) H	53/142 (37.3) L	14/96 (14.6)	8/49 (16.3)	6/47 (12.8)
C	76.3	79/117 (67.5) H	48/115 (41.7) H	16/91 (17.6)	6/40 (15.0)	10/51 (19.6)
D	76.5	87/158 (55.1) H	72/158 (45.6) H	28/125 (22.4)	14/62 (22.6)	14/63 (22.2)
E	77.3	79/164 (48.2) H	55/163 (33.7) L	21/111 (18.9)	4/47 (8.5)	17/64 (26.6)
F	76.5	85/194 (43.8) L	91/192 (47.4) H	34/147 (23.1)	13/70 (18.6)	21/77 (27.3)
G	78.6	109/239 (45.6) L	97/243 (39.9) L	41/170 (24.1)	19/74 (25.7)	22/96 (22.9)
H	77.6	80/172 (46.5) L	69/172 (40.1) H	36/121 (29.8)	18/63 (28.6)	18/58 (31.0)
Total	77.0	677/1357 (49.9)	539/1364 (39.5)	210/984 (21.3)	88/459 (19.2)	122/525 (23.2)

Notes: H = high (top four areas); L = low (lower four areas); * Number of “participate”/study population for community level social participation; **^†^** Number of “tend to trust”/study population for community level generalized trust.

**Table 3:** Association of community and individual level social capital with composite outcome: results from multilevel logistic regression models.

Social Capital	Model 1 *		Model 2 ^†^		Model 3 ^‡^	
	OR (95% CI)	*p*-Value	OR (95% CI)	*p*-Value	OR (95% CI)	*p*-Value
**Total (*n* = 984)**						
**Social participation**						
**Community level ^§^**						
High	1.00		1.00		1.00	
Low	1.34 (0.95–1.89)	0.10	1.26 (0.88–1.78)	0.20	1.25 (0.88–1.78)	0.22
**Individual level**						
Participate			1.00		1.00	
Not participate			2.08 (1.47–2.96)	< 0.001	1.97 (1.38–2.81)	< 0.001
**Generalized trust**						
**Community level ^||^**						
High	1.00		1.00		1.00	
Low	0.65 (0.47–0.92)	0.01	0.62 (0.44–0.87)	0.006	0.62 (0.43–0.87)	0.007
**Individual level**						
Tend to trust			1.00		1.00	
Tend to be careful			1.33 (0.94–1.89)	0.11	1.31 (0.92–1.88)	0.13
**Men (*n* = 459)**						
**Social participation**						
**Community level**						
High	1.00		1.00		1.00	
Low	1.38 (0.83–2.30)	0.22	1.30 (0.76–2.21)	0.34	1.30 (0.75–2.23)	0.35
**Individual level**						
Participate			1.00		1.00	
Not participate			2.40 (1.40–4.09)	0.001	2.33 (1.36–4.00)	0.002
**Generalized trust**						
**Community level**						
High	1.00		1.00		1.00	
Low	0.65 (0.39–1.07)	0.09	0.57 (0.33–0.98)	0.04	0.55 (0.32–0.96)	0.04
**Individual level**						
Tend to trust			1.00		1.00	
Tend to be careful			2.16 (1.25–3.74)	0.006	2.22 (1.27–3.90)	0.005
**Women (*n* = 525)**						
**Social participation**						
**Community level**						
High	1.00		1.00		1.00	
Low	1.31 (0.82–2.09)	0.26	1.22 (0.76–1.96)	0.41	1.23 (0.76–1.99)	0.40
**Individual level**						
Participate			1.00		1.00	
Not participate			1.87 (1.16–3.02)	0.01	1.72 (1.05–2.81)	0.03
**Generalized trust**						
**Community level**						
High	1.00		1.00		1.00	
Low	0.66 (0.41–1.04)	0.08	0.64 (0.40–1.02)	0.06	0.63 (0.39–1.01)	0.06
**Individual level**						
Tend to trust			1.00		1.00	
Tend to be careful			0.93 (0.58–1.49)	0.76	0.92 (0.57–1.49)	0.75

Notes: OR = odds ratio; CI = confidence interval; * Model 1. Community level social capital adjusted for age (continuous), and sex; ^†^ Model 2. Community level and individual level social capital adjusted for age (continuous), and sex; ^‡^ Model 3. Community level and individual level social capital adjusted for age (continuous), sex, marital status, educational attainment, number of people living together, and self-rated health; ^§^ Top four areas (high) and lower four areas (low) for social participation at community level; ^||^ Top four areas (high) and lower four areas (low) for generalized trust at community level.

## Supplementary Materials

The following are available online at www.mdpi.com/1660-4601/13/9/860/s1, Table S1: Association of community and individual level social capital with decline in ADL (excluding deaths): results from multilevel logistic regression models; Table S2: Association of community and individual level social capital with composite outcome excluding those who had outcome in the first year of the follow-up period: results from multilevel logistic regression models; Table S3: Association of community and individual level social capital with composite outcome considering a history of major diseases: results from multilevel logistic regression models.

## Abbreviations

The following abbreviations are used in this manuscript:

ADL	Activities of Daily Living
OR	odds ratio
CI	confidence interval
