# Supplementary material for: Relationships of Community and Individual Level Social Capital with Activities of Daily Living and Death by Gender
Source: Int J Environ Res Public Health. 2016 Aug 29;13(9):860. doi: 10.3390/ijerph13090860 (PMC5036693; doi:10.3390/ijerph13090860)
Supplement: Supplementary file 1 [file ijerph-13-00860-s001.pdf]

Supplementary Materials: Relationships of Community and Individual Level Social Capital with Activities of Daily Living and Death by Gender

Haruhiko Imamura, Tsuyoshi Hamano, Takehiro Michikawa, Fujimi Takeda-Imai, Takahiro Nakamura, Toru Takebayashi and Yuji Nishiwaki

Table S1. (Sensitivity analyses 1). Association of community and individual level social capital with decline in ADL (excluding deaths): results from multilevel logistic regression models.

Social Capital	Model 1 *		Model 2 †		Model 3 ‡	
	OR (95% CI)	*p*-Value	OR (95% CI)	*p*-Value	OR (95% CI)	*p*-Value
**(Total (*n* = 952))**						
**Social participation**						
**Community level §**						
High	1.00		1.00		1.00	
Low	1.41 (0.97–2.05)	0.07	1.34 (0.92–1.96)	0.12	1.35 (0.92–1.97)	0.13
**Individual level**						
Participate			1.00		1.00	
Not participate			2.08 (1.43–3.03)	<0.001	1.96 (1.34–2.88)	0.001
**Generalized trust**						
**Community level ¦¦**						
High	1.00		1.00		1.00	
Low	0.69 (0.48–0.99)	0.05	0.65 (0.45–0.95)	0.03	0.66 (0.45–0.96)	0.03
**Individual level**						
Tend to trust			1.00		1.00	
Tend to be careful			1.38 (0.94–2.01)	0.10	1.36 (0.93–1.99)	0.12
**(Men (*n* = 441))**						
**Social participation**						
**Community level**						
High	1.00		1.00		1.00	
Low	1.72 (0.97–3.05)	0.06	1.64 (0.91–2.97)	0.10	1.60 (0.89–2.89)	0.12
**Individual level**						
Participate			1.00		1.00	
Not participate			2.42 (1.36–4.32)	0.003	2.32 (1.32–4.09)	0.003
**Generalized trust**						
**Community level**						
High	1.00		1.00		1.00	
Low	0.73 (0.42–1.27)	0.27	0.64 (0.36–1.16)	0.15	0.66 (0.37–1.19)	0.17
**Individual level**						
Tend to trust			1.00		1.00	
Tend to be careful			2.34 (1.28–4.30)	0.006	2.27 (1.23–4.21)	0.009
**(Women (*n* = 511))**						
**Social participation**						
**Community level**						
High	1.00		1.00		1.00	
Low	1.23 (0.75–2.02)	0.41	1.15 (0.70–1.91)	0.57	1.18 (0.71–1.97)	0.52
**Individual level**						
Participate			1.00		1.00	
Not participate			1.88 (1.13–3.12)	0.02	1.75 (1.04–2.95)	0.04
**Generalized trust**						
**Community level**						
High	1.00		1.00		1.00	
Low	0.66 (0.40–1.08)	0.10	0.64 (0.39–1.05)	0.08	0.63 (0.38–1.04)	0.07
**Individual level**						
Tend to trust			1.00		1.00	
Tend to be careful			0.95 (0.57–1.56)	0.83	0.96 (0.58–1.59)	0.86

Notes: OR = odds ratio; CI = confidence interval; * Model 1. Community level social capital adjusted for age (continuous), and sex; † Model 2. Community level and individual level social capital adjusted for age (continuous), and sex; ‡ Model 3. Community level and individual level social capital adjusted for age (continuous), sex, marital status, educational attainment, number of people living together, and self-rated health; § Top four areas (high) and lower four areas (low) for social participation at community level; ¦¦ Top four areas (high) and lower four areas (low) for generalized trust at community level.

Table S2. (Sensitivity analyses 2). Association of community and individual level social capital with composite outcome excluding those who had outcome in the first year of the follow-up period: results from multilevel logistic regression models.

Social Capital	Model 1 *		Model 2 †		Model 3 ‡	
	OR (95% CI)	*p*-Value	OR (95% CI)	*p*-Value	OR (95% CI)	*p*-Value
**(Total (*n* = 902))**						
**Social participation**						
**Community level §**						
High	1.00		1.00		1.00	
Low	1.18 (0.78–1.78)	0.43	1.13 (0.75–1.71)	0.55	1.13 (0.75–1.72)	0.56
**Individual level**						
Participate			1.00		1.00	
Not participate			1.55 (1.02–2.35)	0.04	1.55 (1.02–2.35)	0.04
**Generalized trust**						
**Community level ǁ**						
High	1.00		1.00		1.00	
Low	0.57 (0.38–0.85)	0.006	0.54 (0.36–0.82)	0.004	0.54 (0.36–0.83)	0.004
**Individual level**						
Tend to trust			1.00		1.00	
Tend to be careful			1.38 (0.91–2.10)	0.13	1.38 (0.91–2.12)	0.13
**(Men (*n* = 423))**						
**Social participation**						
**Community level**						
High	1.00		1.00		1.00	
Low	1.22 (0.66–2.25)	0.54	1.18 (0.63–2.21)	0.61	1.22 (0.65–2.29)	0.55
**Individual level**						
Participate			1.00		1.00	
Not participate			1.86 (0.99–3.48)	0.05	1.83 (0.98–3.44)	0.06
**Generalized trust**						
**Community level**						
High	1.00		1.00		1.00	
Low	0.50 (0.27–0.94)	0.03	0.44 (0.23–0.84)	0.01	0.42 (0.21–0.81)	0.01
**Individual level**						
Tend to trust			1.00		1.00	
Tend to be careful			2.82 (1.41–5.63)	0.003	3.00 (1.48–6.08)	0.002
**(Women (*n* = 479))**						
**Social participation**						
**Community level**						
High	1.00		1.00		1.00	
Low	1.15 (0.66–1.99)	0.62	1.09 (0.62–1.90)	0.77	1.07 (0.61–1.88)	0.82
**Individual level**						
Participate			1.00		1.00	
Not participate			1.38 (0.79–2.41)	0.26	1.38 (0.78–2.43)	0.27
**Generalized trust**						
**Community level**						
High	1.00		1.00		1.00	
Low	0.62 (0.36–1.06)	0.08	0.62 (0.36–1.07)	0.08	0.61 (0.35–1.06)	0.08
**Individual level**						
Tend to trust			1.00		1.00	
Tend to be careful			0.85 (0.49–1.48)	0.57	0.85 (0.49–1.49)	0.58

Notes: OR = odds ratio; CI = confidence interval; * Model 1. Community level social capital adjusted for age (continuous), and sex; † Model 2. Community level and individual level social capital adjusted for age (continuous), and sex; ‡ Model 3. Community level and individual level social capital adjusted for age (continuous), sex, marital status, educational attainment, number of people living together, and self-rated health; § Top four areas (high) and lower four areas (low) for social participation at community level; ǁ Top four areas (high) and lower four areas (low) for generalized trust at community level.

Table S3. (Sensitivity analyses 3). Association of community and individual level social capital with composite outcome considering a history of major diseases: results from multilevel logistic regression models.

Social Capital	Model 1 *		Model 2 †		Model 3 ‡	
	OR (95% CI)	*p*-Value	OR (95% CI)	*p*-Value	OR (95% CI)	*p*-Value
**(Total (*n* = 439))**						
**Social participation**						
**Community level** §						
High	1.00		1.00		1.00	
Low	1.04 (0.56–1.92)	0.91	1.03 (0.58–1.85)	0.92	1.05 (0.58–1.89)	0.88
**Individual level**						
Participate			1.00		1.00	
Not participate			2.12 (1.23–3.66)	0.007	2.11 (1.21–3.69)	0.009
**Generalized trust**						
**Community level** ¦¦						
High	1.00		1.00		1.00	
Low	0.74 (0.40–1.37)	0.34	0.65 (0.36–1.17)	0.15	0.69 (0.38–1.23)	0.21
**Individual level**						
Tend to trust			1.00		1.00	
Tend to be careful			1.23 (0.72–2.08)	0.45	1.15 (0.67–1.98)	0.62
**(Men (*n* = 198))**						
**Social participation**						
**Community level**						
High	1.00		1.00		1.00	
Low	1.02 (0.42–2.43)	0.97	0.96 (0.37–2.53)	0.94	1.12 (0.43–2.92)	0.81
**Individual level**						
Participate			1.00		1.00	
Not participate			2.04 (0.85–4.90)	0.11	1.93 (0.78–4.79)	0.16
**Generalized trust**						
**Community level**						
High	1.00		1.00		1.00	
Low	0.56 (0.23–1.36)	0.20	0.44 (0.16–1.20)	0.11	0.46 (0.17–1.20)	0.11
**Individual level**						
Tend to trust			1.00		1.00	
Tend to be careful			3.29 (1.31–8.22)	0.01	3.67 (1.39–9.67)	0.009
**(Women (*n* = 241))**						
**Social participation**						
**Community level**						
High	1.00		1.00		1.00	
Low	1.17 (0.58–2.36)	0.66	1.11 (0.54–2.26)	0.78	1.05 (0.51–2.17)	0.90
**Individual level**						
Participate			1.00		1.00	
Not participate			2.09 (1.04–4.23)	0.04	2.15 (1.03–4.48)	0.04
**Generalized trust**						
**Community level**						
High	1.00		1.00		1.00	
Low	0.92 (0.46–1.85)	0.81	0.86 (0.42–1.77)	0.69	0.89 (0.43–1.85)	0.76
**Individual level**						
Tend to trust			1.00		1.00	
Tend to be careful			0.68 (0.34–1.37)	0.28	0.62 (0.30–1.27)	0.19

Notes: OR = odds ratio; CI = confidence interval; * Model 1. Community level social capital adjusted for age (continuous), and sex; † Model 2. Community level and individual level social capital adjusted for age (continuous), and sex; ‡ Model 3. Community level and individual level social capital adjusted for age (continuous), sex, marital status, educational attainment, number of people living together, self-rated health, and medical history of major diseases (stroke, myocardial infarction/angina, diabetes, Parkinson's disease, or cancer); § Top four areas (high) and lower four areas (low) for social participation at community level; ¦¦ Top four areas (high) and lower four areas (low) for generalized trust at community level.

© 2016 by the authors; licensee MDPI, Basel, Switzerland. This article is an open access article distributed under the terms and conditions of the Creative Commons by Attribution (CC-BY) license (http://creativecommons.org/licenses/by/4.0/).
